# Association between 23 drugs and Parkinson's disease: A two‐sample Mendelian randomization study

**DOI:** 10.1002/brb3.3225

**Published:** 2023-08-31

**Authors:** Zhixin Xie, Haobin Zhou, Ziyu Qiu, Zhongxi Fan, Weisheng Yang, Jingbai Zhang, Yezhong Wang, Yongyi Ye

**Affiliations:** ^1^ Department of Clinical Medicine The Second Clinical College of Guangzhou Medical University Guangzhou Guangdong China; ^2^ Department of Clinical Medicine The First Clinical College of Guangzhou Medical University Guangzhou Guangdong China; ^3^ Department of Clinical Medicine The Third Clinical College of Guangzhou Medical University Guangzhou Guangdong China; ^4^ Department of Nursing The Nursing College of Guangzhou Medical University Guangzhou Guangdong China; ^5^ Department of Neurosurgery The Second Affiliated Hospital of Guangzhou Medical University Guangzhou Guangdong China

**Keywords:** drug, Parkinson's disease, two‐sample Mendelian randomization

## Abstract

**Background:**

Parkinson's disease (PD) is a common degenerative nervous system disease. At present, there are certain limitations in various treatment options aimed at preventing or delaying the progression of PD. Therefore, the exploration of new drugs for PD is beneficial. Mendelian randomization (MR) analysis can be used to explore the association between drugs and diseases. In this study, MR analysis was adopted to investigate the causal relationship between 23 drugs and PD. These drugs have been approved for the treatment of different diseases, such as salicylic acid and derivatives (collectively called salicylates, e.g., aspirin, used for fever and pain relief), antithrombotic agents (e.g., warfarin, aspirin, used for preventing thrombotic events).

**Methods:**

The GWAS data for the 23 drugs were obtained from the UK Biobank (UKB) project, while the GWAS data for PD were sourced from FinnGen. Single‐Nucleotide Polymorphisms (SNPs) were selected as instrumental variables (IVs). We first performed a series of quality control steps (including MR‐PRESSO) to select the appropriate SNPs. Two‐sample MR analysis was performed using five different methods, including inverse variance weighting (IVW) with random‐effects model, weighted median, MR‐Egger, simple model, and weighted model. At the same time, sensitivity analysis was carried out using the MR‐Egger and Cochran's *Q* test to ensure the authenticity and reliability of the results.

**Results:**

In MR‐PRESSO, salicylates and antithrombotic agents showed statistically significant associations with PD, respectively. In the main MR analysis (IVW), there was a negative causal relationship between salicylates and PD (OR = 0.73, 95% CI = 0.54–0.98, *p* = .039). Similarly, there was a negative causal relationship between antithrombotic agents and PD (OR = 0.70, 95%CI = 0.52–0.96, *p* = .027). No statistically significant association was found between the remaining 21 drugs and PD.

**Conclusion:**

This MR study demonstrated that salicylates and antithrombotic agents can reduce the risk of PD, thus providing a novel avenue for future drug exploration in PD.

## Graphical abstract

Two‐Sample Mendelian Randomization analysis was adopted in this study to explore the causal relationship between 23 drugs and Parkinson's disease (PD). The results of this study indicate a negative causal relationship between Salicylic acid and derivatives and PD, as well as Antithrombotic agents and PD. No statistically significant association was found between the remaining 21 drugs and PD.

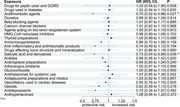

## 1 INTRODUCTION

With the intensifying aging population, the incidence of noncommunicable neurodegenerative diseases is likely to continue rising, posing significant societal pressures (GBD 2016 Neurology Collaborators, 2019). Among them, Neurodegenerative diseases (ND) are commonly observed, particularly among the elderly population. Aging is a primary risk factor for ND, as it influences the occurrence of ND through various mechanisms such as DNA damage, mitochondrial dysfunction, and inflammation. Neurodegeneration and the associated decline in cognitive abilities significantly impact human health and quality of life (Hou et al., 2019). ND encompasses a range of conditions, including Alzheimer's disease (AD), Parkinson's disease (PD), and progressive supranuclear palsy (PSP), among others. Among these, PD ranks as the second most common neurodegenerative disease, following closely behind AD (Erkkinen et al., 2018). PD arises from the depletion and loss of substantia nigra neurons in the human brain, causing a paucity of dopamine in the striatum, which triggers a cascade of neurodegenerative events (Poewe et al., 2017). The primary clinical manifestations of PD consist of movement symptoms dominated by tremors and bradykinesia, as well as nonmotor symptoms such as depression, anxiety, and cognitive decline (Hayes, 2019). Current treatments have limitations in preventing or delaying PD progression (Bloem et al., 2021; Jankovic et al., 2020). Due to various factors, many drug trials for PD have not achieved ideal outcomes (Vijiaratnam et al., 2021). Hence, exploring therapeutic drugs for PD through new methods may be beneficial.

Mendelian randomization (MR) analysis, which employs genetic variation as instrumental variables (IVs), is a method for inferring causal relationships between exposure and outcome (Davey Smith & Hemani, 2014). MR analysis can overcome the effects of confounding factors, such as behavioral and environmental factors (Burgess et al., 2012; Smith & Ebrahim, 2004). Moreover, it can provide reliable evidence for causal relationships between risk factors and diseases, while guiding the direction of clinical trials and drug development (Burgess et al., 2012; Davies et al., 2018).

This study employs two‐sample MR to assess causal relationships between multiple drugs and PD, utilizing the latest drug genome‐wide association study (GWAS) data that covers a wide range of populations, and evaluating the reliability of MR results. The results of this study provide new evidence supporting the exploration of causal relationships between multiple drugs and PD, and at the same time, it may provide direction for future drug trials.

## 2 MATERIALS AND METHODS

### 2.1 Study design and basic assumptions of MR

This is an MR study investigating the causal relationship between 23 drugs and Parkinson's disease (PD). The MR study relies on the strict adherence to three assumptions: (1) the relevance assumption, where IVs should be strongly associated with the exposure; (2) the independence assumption, where the effect of IVs on the outcome can only be mediated through the exposure; and (3) the exclusion restriction assumption, where IVs should not have a direct association with the outcome (Emdin et al., 2017). Moreover, we have followed the recommendations of Strengthening the Reporting of Observational Studies in Epidemiology Using Mendelian Randomization (STROBE‐MR) to ensure the transparency and replicability of our study (see the STROBE‐MR CHECKLIST in Supplementary File for details) (Skrivankova et al., 2021).

The R package TwoSampleMR was utilized for conducting the MR analysis, while the R package MR‐PRESSO was employed for performing MR‐PRESSO. All the aforementioned analyses were carried out on R software version 4.2.1.

### 2.2 GWAS summary statistics

Mendelian randomization (MR) used data from a genome‐wide association study (GWAS) of 23 drugs as exposures and utilized them to quantify the risk of future drug use. Our study sample was obtained from the UK Biobank (UKB) project (https://www.ukbiobank.ac.uk/), in which 502,616 participants (about 54% females) had medical records at their first UKB assessment visit (Wu et al., 2019). The UKB contains 6745 medicines which were classified into 23 categories and underwent multiple quality checks to generate pooled GWAS data. We obtained the GWAS data for these 23 drugs, including salicylic acid and derivatives, antithrombotic agents, opioids, antidepressant, antihypertensives, etc. For the outcome dataset, GWAS data for PD were obtained from FinnGen (https://Finngen.gitbook.io/documentation/) and included 3767 cases and 338,732 controls. More summary statistics about the exposure and outcome are presented in the Supplementary Files S1 and S2.

In this study, the sample for the GWAS of 23 drugs primarily comes from the UK Biobank (UKB), while the sample for the GWAS of Parkinson's disease (PD) comes from FinnGen. It implied that the GWAS data for exposure and outcomes were derived from two primarily independent samples, making sample overlap and its impact on the study results potentially negligible. In addition, both samples consist of individuals of European ancestry, so it can be considered that there was a similarity of the genetic variant‐exposure associations between the exposure and outcome samples.

### 2.3 SNP selection

The MR framework uses independent instrumental SNPs as exposed instrumental variables (IVs) to estimate and test the causal relationships with outcomes. To satisfy the three strict assumptions mentioned earlier (Emdin et al., 2017), we performed a series of quality control steps to select suitable SNPs. We selected SNPs with a genome‐wide association (*p* < 5E‐08), with independent inheritance (*r* ^2^ < 0.01), and without linkage disequilibrium (LD) in summary statistics. We also calculated the *F* statistic for each exposure to avoid bias due to weak genetic instruments. IVs with an *F* statistic of less than 10 were excluded and were often labeled as “weak instruments” (Cui et al., 2021). The calculation formula is as follows (Kurilshikov et al., 2021):

$$\frac{r^{2}\left(N-2\right)}{\left(1-r^{2}\right)}.$$

(*N*: The sample size and *r* ^2^: the variance explained by IVs)

Since subsequent analysis required at least four SNPs as instrumental variables, we relaxed the *p* value threshold to *p* < 5E‐07 for screening drug GWAS with insufficient instrumental variables after normalization. If that is not enough, the threshold was further relaxed to *p* < 5E‐06. Moreover, the outliers of missing data were excluded.

### 2.4 MR analysis

After identifying the genetic instruments for each exposure, genetic variants associated with drugs were selected as genetic instruments for each exposure. We first applied MR‐PRESSO to detect and correct for any outliers reflecting likely pleiotropic biases for all reported results (Verbanck et al., 2018). If corrections were present, the corrected *p* value was used. Two‐sample MR analysis was performed using five different methods, including inverse variance weighted (IVW) with random‐effects model, weighted median, MR‐Egger, simple mode, and weighted mode (Sun et al., 2022). Each method makes different assumptions on the validity of IVs, but the IVW method is generally considered the most reliable. IVW was used as our principal model, which accounts for heterogeneity in the variant‐specific causal estimates (Burgess et al., 2019). The other methods were used as complementary or to observe if their results were consistent with the direction of IVW. Finally, a forest plot was used for visualization.

### 2.5 Sensitivity analyses

In this study, sensitivity analyses were performed using different methods. We conducted the MR‐Egger regression to evaluate the possibility of horizontal pleiotropy. The intercept term of the MR‐Egger regression shows the mean pleiotropic effect IV (Bowden et al., 2015). To assess the heterogeneity of the effects we used Cochran's *Q* test with IVW and MR‐Egger, and *p* < .05 determined by Cochran's *Q* test was considered heterogeneous (Haycock et al., 2016). The leave‐one‐out sensitivity analysis was performed to test the robustness of the association results by removing studies individually.

## 3 RESULT

### 3.1 Characteristics of the selected SNPs

Two‐sample MR analysis was applied to explore the relationships between 23 drugs and PD, including salicylic acid and derivatives (collectively called salicylates), antithrombotic agents, vasodilators used in cardiac disease, opioids, antidepressant, antihypertensives, and so on. Since there were no SNPs with a *p* value less than 5E‐08 for some drugs and PD, we broadened the threshold to 5E‐07 (vasodilators used in cardiac disease, opioids, antidepressant) and 5E‐06 (antihypertensives) to select qualified instrumental variables. The SNP was removed if the EAF was not successfully matched. Finally, 101 SNPs of thyroid preparations were included. For salicylates, 9 SNPs were selected, and 10 SNPs for antithrombotic agents were included. All included SNPs adhere to the criteria of independent inheritance (*r* ^2^ < 0.01), no linkage disequilibrium (LD), and *F* > 10 (“weak instruments ” were excluded). For the inclusion of further exposed SNPs, see the Supplementary File S3 for details.

### 3.2 MR analysis

**FIGURE 1:**
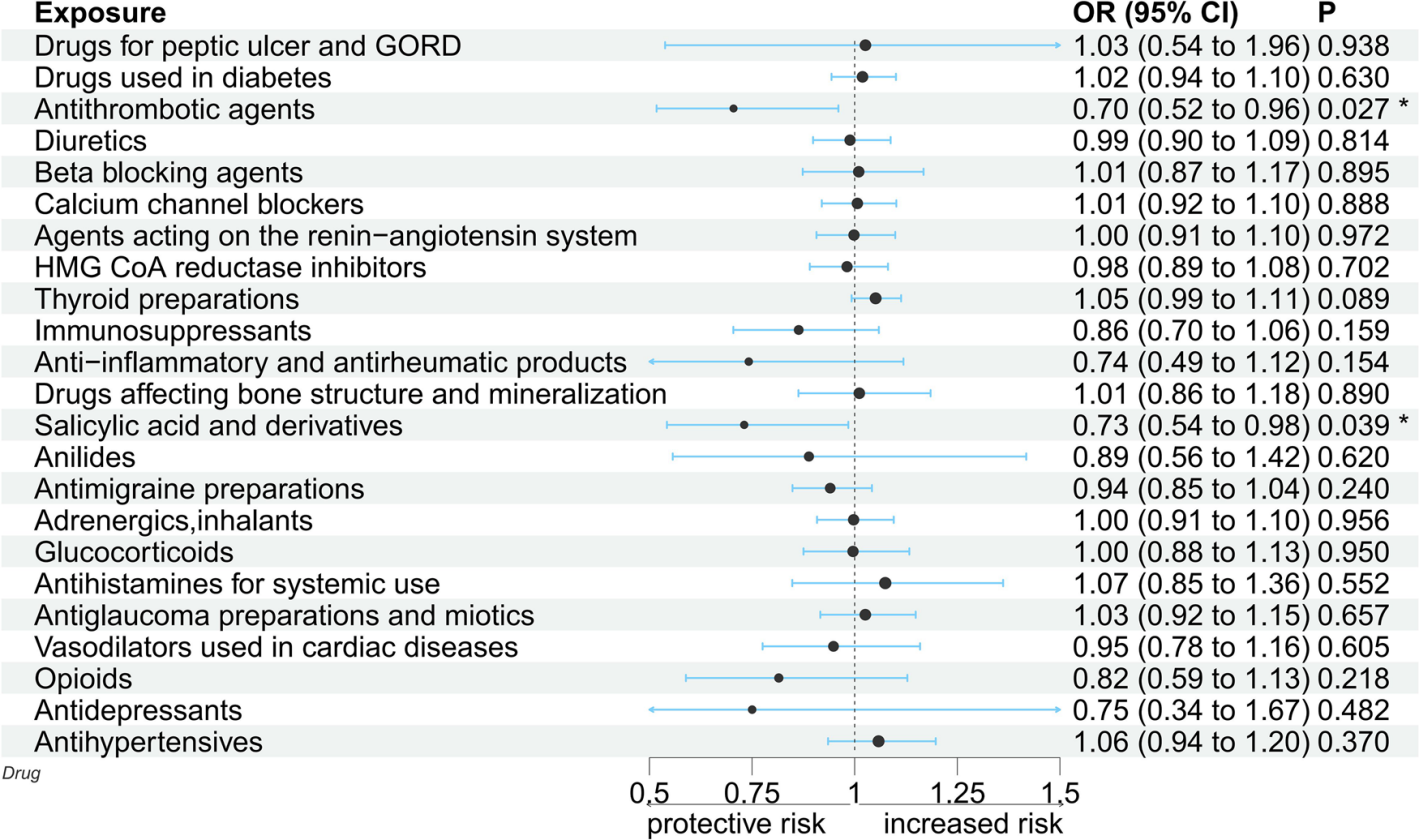
Summary of the MR estimation in IVW. OR: odds ratio; CI: confidence interval; P: *p* value of MR.

After identifying and removing abnormal SNPs, the results of MR‐PRESSO described a statistically significant causal relationship between salicylates (*p* = .017), antithrombotic agents (*p* = .022), and PD (Supplementary File S3). In the MR analysis, IVW showed that both two factors were negatively associated with the incidence of PD. As shown in Figure 1 and the Supplementary File S3, salicylates reduced the risk of PD (OR = 0.73, 95% CI = 0.54–0.98, *p* = .039), and the directions of β values of the remaining four methods are consistent with IVW. In addition, MR analysis also demonstrated the therapeutic effect of antithrombotic agents on PD (OR = 0.70, 95%CI = 0.52–0.96, *p* = .027), and the direction of β value of the five MR methods was consistent, which confirmed the robustness and reliability of the results. However, no causal relationship was found between other drugs and PD. Details are listed in the Supplementary File S3.

### 3.3 Sensitivity analyses

**FIGURE 2:**
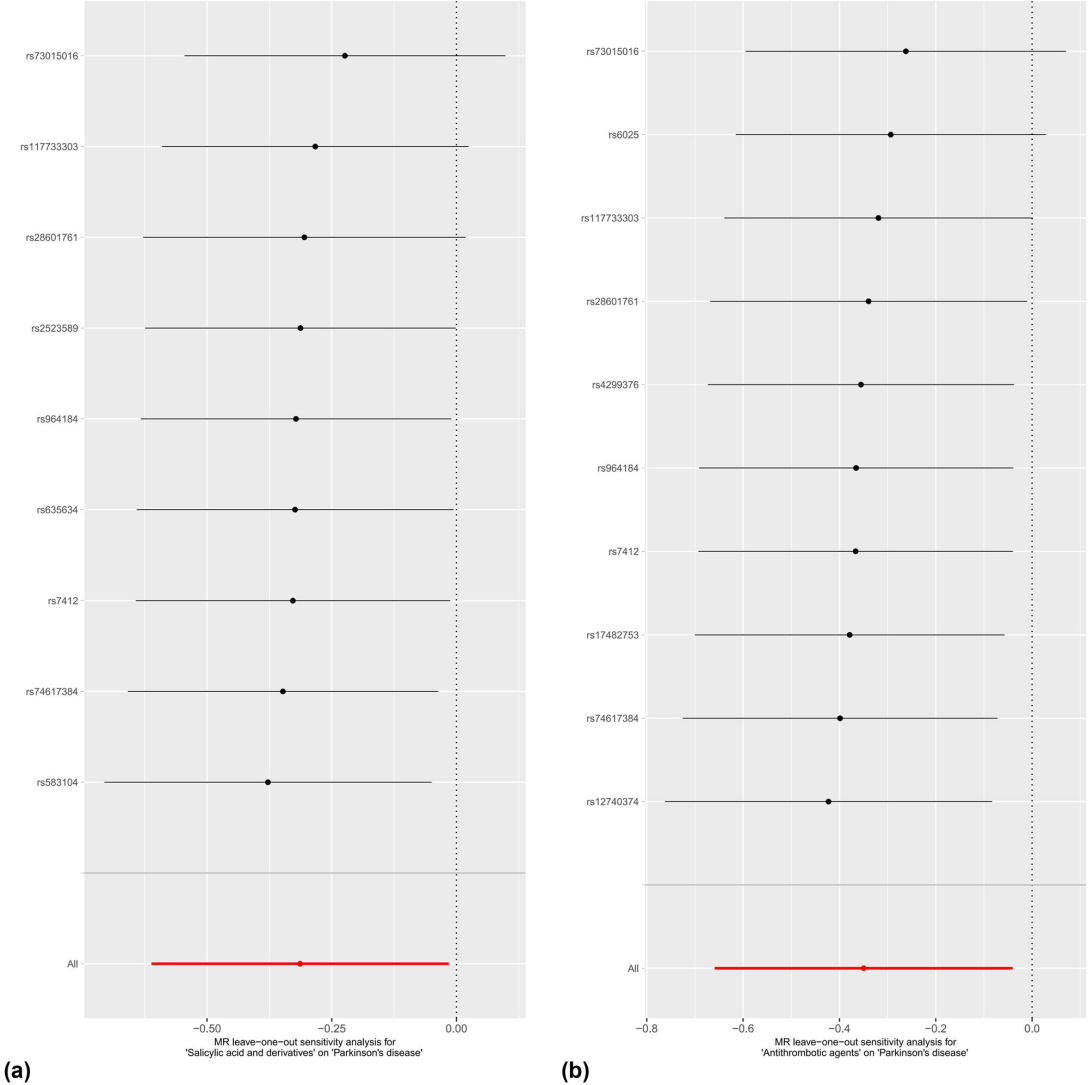
(a) MR leave‐one‐out analysis for salicylic acid and derivatives and PD. (b) MR leave‐one‐out analysis for antithrombotic agents and PD.

**FIGURE 3:**
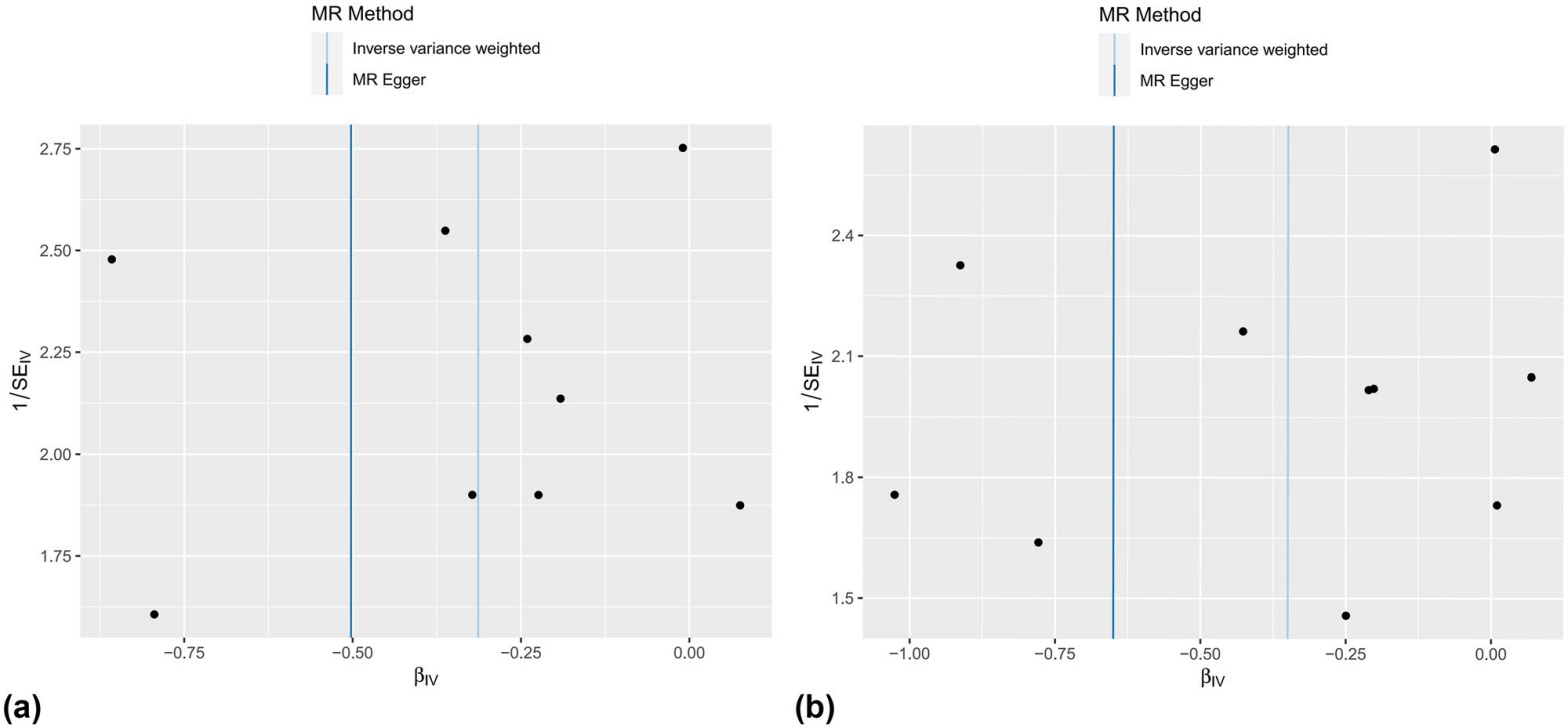
(a) Funnel plots of the association between salicylic acid and derivatives and PD. (b) Funnel plots of the association between antithrombotic agents and PD. The dark blue line represents MR‐Egger and the light blue line represents IVW.

**FIGURE 4:**
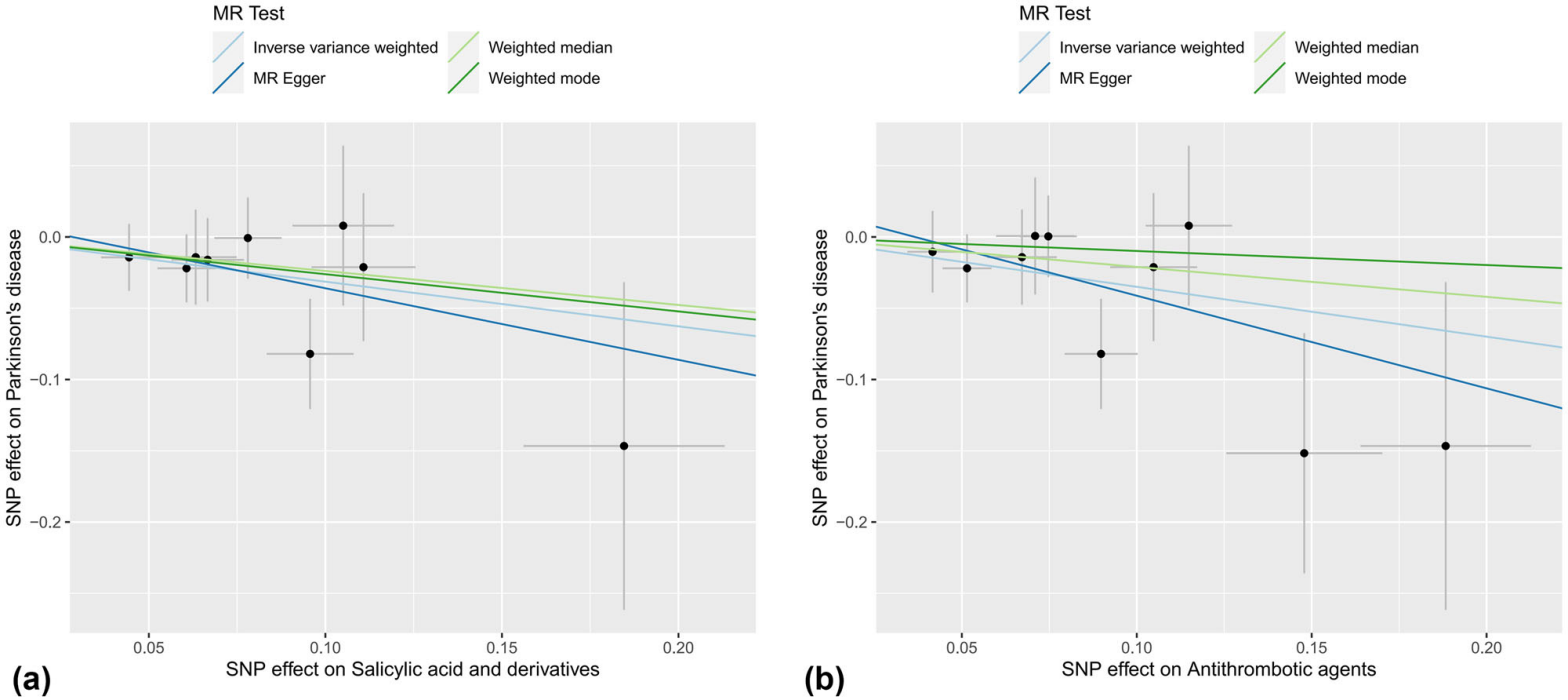
(a) Scatterplot analysis for salicylic acid and derivatives and PD. (b) Scatterplot analysis for antithrombotic agents and PD. The dark blue line represents MR‐Egger, the light blue line represents IVW, the dark green line represents the weighted mode, and the light green line represents the weighted median.

We performed MR‐Egger intercept tests to evaluate the possibility of horizontal pleiotropy, and no significant horizontal pleiotropy was found in the association of salicylates with PD (intercept = 0.014, *p* = .707). Similarly, MR‐Egger showed that there was no horizontal pleiotropy between antithrombotic agents and PD (*p* = .499), which further proved the reliability of our causal inference results. Bias from horizontal pleiotropies could be largely ruled out by using leave‐one‐out analysis, which shows that our MR results are stable and not driven by any single SNP (Figure 2). In Cochran's *Q* test for heterogeneity, both IVW (*Q* = 3.787, *p* = .876) and MR‐Egger (*Q* = 3.633, *p* = .821) showed there was no heterogeneity among SNPs of salicylates. In addition, Cochran's *Q* test between antithrombotic agents and PD also shows no heterogeneity (*p* > .05), indicating that the IVW results of the multiplicative random‐effects method should be the first choice. Funnel plot and scatter plot were used for visualization (Figures 3 and 4).

Finally, the summary results of the MR estimation and sensitivity analysis for all drugs and PD are given in the Supplementary File S3. Overall, the significance of the causal relationship between salicylates and PD, as well as antithrombotic agents and PD, was repeated by MR‐PRESSO and IVW, and passed the pleiotropic test. In conclusion, it was found in our MR study that salicylates and antithrombotic agents can reduce the risk of PD.

## 4 DISCUSSION

Based on 23 exposure GWAS datasets and 1 outcome GWAS dataset, this MR analysis examined the relationship between 23 drugs and PD and found a statistically negative correlation between salicylates and PD, as well as between antithrombotic agents and PD, indicating that both drugs can reduce the risk of PD. No significant association was found between the other 21 drugs and PD. The results of this analysis provide support for the causal relationship between salicylates and PD, and antithrombotic agents and PD, and offer new insights for the development of PD drug treatments.

Aspirin (acetylsalicylic acid), a derivative of salicylic acid, has been widely used as a medication since its inception in the late nineteenth century (Hybiak et al., 2020). Currently, numerous studies suggest that aspirin may be advantageous in the treatment of PD (Gabbert et al., 2022; Rangasamy et al., 2019; San Luciano et al., 2020). Regular use of aspirin has been associated with a lowered risk of PD in LRRK2 mutation carriers (San Luciano et al., 2020). The utilization of aspirin may potentially postpone the onset age of PD (Gabbert et al., 2022). Aspirin is capable of boosting dopamine production by increasing the level of tyrosine hydroxylase in the mouse substantia nigra, which may be linked to its ability to trigger the activation of cAMP response element‐binding protein in dopaminergic neurons (Rangasamy et al., 2019). Another variant of salicylates is sodium salicylate, which has demonstrated promising preventive effects against MPTP‐induced dopaminergic toxicity and neurotoxicity in mice (Ferger et al., 1999; Thrash‐Williams et al., 2016).

Oxidative stress and inflammation are key factors in the progression of PD. When there is oxidative stress within cells, the accumulation of reactive oxygen species (ROS) can lead to oxidative damage, thus damaging neurons (Subramaniam & Chesselet, 2013; Trist et al., 2019). Inflammation is also commonly present in the progression of PD, and various pathological and physiological processes such as activation of microglial cells and regulation of the immune system can cause inflammation, thereby affecting the nervous system (Pajares et al., 2020; Tansey et al., 2022). Oxidative stress can cause oxidative damage, stimulate the production of inflammation, and the presence of inflammatory factors can exacerbate oxidative damage, leading to a vicious cycle (Pajares et al., 2020).

Aspirin inhibits the enzyme activity of cyclooxygenase (COX), which affects the generation of prostaglandins (PGs), thereby inhibiting inflammation (Bindu et al., 2020). At the same time, aspirin also has an antioxidative stress effect, which can prevent oxidative damage (Chen et al., 2020; Paseban et al., 2019). Therefore, aspirin may affect PD by influencing these two factors. However, it should be noted that the conclusions of current studies are not consistent. According to a retrospective investigation, the utilization of aspirin has been purportedly linked with a considerably heightened vulnerability to PD (Chen et al., 2021). Also, there was no statistically significant correlation between low‐dose aspirin and the risk of multiple system atrophy (MSA, a rare atypical form of PD) (Starhof et al., 2020). A case‐control study also found no statistically significant association between the long‐term use of aspirin and the incidence of PD (Becker et al., 2011). Therefore, more research is needed to determine the relationship between salicylates and PD.

Inhibition of platelet aggregation and thrombin is an important means of anti‐thrombosis. Inflammation promotes platelet aggregation in cerebral microvasculature, while soluble factors released by activated platelets in turn promote the development of inflammation (Page & Pretorius, 2022). Some antiplatelet drugs show promising results in the treatment of PD. Aspirin irreversibly inhibits platelet COX enzyme, thereby inhibiting platelet aggregation mediated by TXA2 (Majithia & Bhatt, 2019). Aspirin also exhibits anti‐inflammatory (Bindu et al., 2020) and antioxidant effects (Chen et al., 2020; Paseban et al., 2019) and has been suggested to be beneficial for PD in some studies (Gabbert et al., 2022; Rangasamy et al., 2019; San Luciano et al., 2020). Cilostazol can increase the expression of Nurr1 in mice, protecting the integrity of dopaminergic neurons and reducing inflammation by suppressing NF‐κB and its downstream effectors TNF‐α and IL‐1β (Hedya et al., 2018). In a 2017 study, dipyridamole was shown to have potential value in protecting neurons in PD patients by reducing levels of lactate dehydrogenase (LDH) in LUHMES cells with overexpression of α‐synuclein (Hollerhage et al., 2017). On the other hand, antiplatelet drugs can also be used to control cardiovascular diseases (common complications of PD) and inhibit thrombus formation, thereby improving patient prognosis (Beura et al., 2022).

Thrombin induces the expression of proinflammatory cytokines such as NO, IL‐1β, IL‐6, and TNF‐α by activating PAR, promoting inflammation, and driving the progression of neurodegenerative diseases (Ebrahimi et al., 2017; Krenzlin et al., 2016). In PD rats induced by rotenone, dabigatran etexilate inhibited thrombin levels and reduced the inflammation level in the substantia nigra, thus exerting a neuroprotective effect (Kandil et al., 2018). In another in vitro experiment, dabigatran inhibited thrombin and reduced the expression of NOX4, iNOS, and SOD in fruit flies, suggesting that thrombin inhibition can reduce oxidative stress, which may be beneficial for PD patients (Johnson et al., 2020). In vitro, heparin induced the formation of new α‐Synuclein complexes, thereby reducing the pathogenicity of amyloid fibrils on neurons (Tao et al., 2022). Direct oral anticoagulants (DOACs) can interrupt thrombin‐induced neurotoxicity and neuroinflammation, thus protecting PD patients (Alaaeddine et al., 2021). These findings suggest that antithrombotic agents have great potential in the treatment of PD.

This article boasts several advantages. To begin with, it draws upon the latest GWAS database for drugs, which encompasses a wide range of populations. Furthermore, this is the first study to confirm the causal relationship between salicylates and PD, as well as between antithrombotic agents and PD, using summary‐level data from large‐scale GWAS databases. As such, our research offers new evidence in support of the prevention and treatment of PD. Finally, when compared to observational studies, the implementation of MR analysis may yield more convincing results, given its ability to reduce the influence of confounding factors and reverse causation.

However, our study also has some limitations. The GWAS data for PD and the 23 drugs are predominantly derived from individuals of European ancestry, with no representation of other ethnic populations. Hence, caution must be exercised when extrapolating our MR analysis outcomes to other groups as it may only be generalizable to European ancestry populations. Furthermore, while two‐sample MR analysis serves as a valuable tool for assessing the causal relationship between drugs and PD, it has certain shortcomings, as it only furnishes estimates of hypothetical causal associations. Consequently, further investigations are imperative to authenticate the direct causal impact of these 23 drugs on PD.

## AUTHOR CONTRIBUTIONS

This study was conceived by Zhixin Xie and Haobin Zhou. Zhixin Xie and Haobin Zhou completed the data collection and analysis. The collation of the results and visualization were done by Zhixin Xie and Haobin Zhou. Ziyu Qiu, Zhongxi Fan, Weisheng Yang, and Jingbai Zhang participated in the completion of the original manuscript. Yongyi Ye and Yezhong Wang supervised the study. All the authors participated in the critical revision and agreed to be responsible for all aspects of the work.

## CONFLICT OF INTEREST STATEMENT

We have no competing interests.

### PEER REVIEW

The peer review history for this article is available at https://publons.com/publon/10.1002/brb3.3225.

## Supporting information

S1: Summary statistics of exposure.
S2: Summary statistics of outcome.
S3: The summary results of MR estimation and sensitivity analysis of 23 drugs and PD.
STROBE‐MR CHECKLIST: Strengthening the Reporting of Observational Studies in Epidemiology Using Mendelian Randomization (STROBE‐MR) CHECKLIST.

Supporting Information

## Data Availability

The summary GWAS dataset of 23 drugs was obtained from the UK Biobank (UKB) project (https://pubmed.ncbi.nlm.nih.gov/31015401/). GWAS dataset for PD was obtained from FinnGen (https://storage.googleapis.com/finngen‐public‐data‐r8/summary_stats/finngen_R8_G6_PARKINSON.gz). The results of the analysis can be found in the manuscript and supplementary documents.

## References

[brb33225-bib-0001] Alaaeddine, R. A. , AlZaim, I. , Hammoud, S. H. , Arakji, A. , Eid, A. H. , Abd‐Elrahman, K. S. , & El‐Yazbi, A. F. (2021). The pleiotropic effects of antithrombotic drugs in the metabolic‐cardiovascular‐neurodegenerative disease continuum: Impact beyond reduced clotting. Clinical Science (London, England: 1979), 135, 1015–1051. 10.1042/CS20201445 33881143

[brb33225-bib-0002] Becker, C. , Jick, S. S. , & Meier, C. R. (2011). NSAID use and risk of Parkinson disease: A population‐based case‐control study. European Journal of Neurology, 18, 1336–1342. 10.1111/j.1468-1331.2011.03399.x 21457177

[brb33225-bib-0003] Beura, S. K. , Panigrahi, A. R. , Yadav, P. , & Singh, S. K. (2022). Role of platelet in Parkinson's disease: Insights into pathophysiology & theranostic solutions. Ageing Research Reviews, 80, 101681. 10.1016/j.arr.2022.101681 35798236

[brb33225-bib-0004] Bindu, S. , Mazumder, S. , & Bandyopadhyay, U. (2020). Non‐steroidal anti‐inflammatory drugs (NSAIDs) and organ damage: A current perspective. Biochemical Pharmacology, 180, 114147. 10.1016/j.bcp.2020.114147 32653589 PMC7347500

[brb33225-bib-0005] Bloem, B. R. , Okun, M. S. , & Klein, C. (2021). Parkinson's disease. Lancet, 397, 2284–2303. 10.1016/S0140-6736(21)00218-X 33848468

[brb33225-bib-0006] Bowden, J. , Davey Smith, G. , & Burgess, S. (2015). Mendelian randomization with invalid instruments: Effect estimation and bias detection through Egger regression. International Journal of Epidemiology, 44, 512–525. 10.1093/ije/dyv080 26050253 PMC4469799

[brb33225-bib-0007] Burgess, S. , Butterworth, A. , Malarstig, A. , & Thompson, S. G. (2012). Use of Mendelian randomisation to assess potential benefit of clinical intervention. Bmj, 345, e7325. 10.1136/bmj.e7325 23131671

[brb33225-bib-0008] Burgess, S. , Davey Smith, G. , Davies, N. M. , Dudbridge, F. , Gill, D. , Glymour, M. M. , Hartwig, F. P. , Holmes, M. V. , Minelli, C. , Relton, C. L. , & Theodoratou, E. (2019). Guidelines for performing Mendelian randomization investigations. Wellcome Open Research, 4, 186. 10.12688/wellcomeopenres.15555.2 32760811 PMC7384151

[brb33225-bib-0009] Chen, C. M. , Tung, Y. T. , Wei, C. H. , Lee, P. Y. , & Chen, W. (2020). Anti‐Inflammatory and reactive oxygen species suppression through aspirin pretreatment to treat hyperoxia‐induced acute lung injury in NF‐kappaB‐luciferase inducible transgenic mice. Antioxidants (Basel), 9, 10.3390/antiox9050429 PMC7278740 32429142

[brb33225-bib-0010] Chen, Y. , Sun, X. , Lin, Y. , Zhang, Z. , Gao, Y. , & Wu, I. X. Y. (2021). Non‐genetic risk factors for Parkinson's disease: An overview of 46 systematic reviews. Journal of Parkinson's Disease, 11, 919–935. 10.3233/JPD-202521 PMC8461677 33814465

[brb33225-bib-0012] Cui, Z. , Feng, H. , He, B. , He, J. , & Tian, Y. (2021). Relationship between serum amino acid levels and bone mineral density: A Mendelian randomization study. Frontiers in Endocrinology (Lausanne), 12, 763538. 10.3389/fendo.2021.763538 PMC8630695 34858335

[brb33225-bib-0013] Davey Smith, G. , & Hemani, G. (2014). Mendelian randomization: Genetic anchors for causal inference in epidemiological studies. Human Molecular Genetics, 23, R89–98. 10.1093/hmg/ddu328 25064373 PMC4170722

[brb33225-bib-0014] Davies, N. M. , Holmes, M. V. , & Davey Smith, G. (2018). Reading Mendelian randomisation studies: A guide, glossary, and checklist for clinicians. Bmj, 362, k601. 10.1136/bmj.k601 30002074 PMC6041728

[brb33225-bib-0015] Ebrahimi, S. , Jaberi, N. , Avan, A. , Ryzhikov, M. , Keramati, M. R. , Parizadeh, M. R. , & Hassanian, S. M. (2017). Role of thrombin in the pathogenesis of central nervous system inflammatory diseases. Journal of Cellular Physiology, 232, 482–485. 10.1002/jcp.25501 27458694

[brb33225-bib-0016] Emdin, C. A. , Khera, A. V. , & Kathiresan, S. (2017). Mendelian randomization. JAMA, 318, 1925–1926. (10.1001/jama.2017.17219 29164242

[brb33225-bib-0017] Erkkinen, M. G. , Kim, M. O. , & Geschwind, M. D. (2018). Clinical neurology and epidemiology of the major neurodegenerative diseases. Cold Spring Harbor perspectives in biology, 10(4), a033118. 10.1101/cshperspect.a033118 28716886 PMC5880171

[brb33225-bib-0018] Ferger, B. , Teismann, P. , Earl, C. D. , Kuschinsky, K. , & Oertel, W. H. (1999). Salicylate protects against MPTP‐induced impairments in dopaminergic neurotransmission at the striatal and nigral level in mice. Naunyn‐Schmiedebergs Archives of Pharmacology, 360, 256–261. 10.1007/s002109900079 10543426

[brb33225-bib-0019] Gabbert, C. , Konig, I. R. , Luth, T. , Kolms, B. , Kasten, M. , Vollstedt, E. J. , Balck, A. , Grunewald, A. , Klein, C. , & Trinh, J. (2022). Coffee, smoking and aspirin are associated with age at onset in idiopathic Parkinson's disease. Journal of Neurology, 269, 4195–4203. 10.1007/s00415-022-11041-x 35235000 PMC9294004

[brb33225-bib-0011] GBD 2016 Neurology Collaborators . (2019). Global, regional, and national burden of neurological disorders, 1990–2016: A systematic analysis for the Global Burden of Disease Study 2016. Lancet Neurology, 18, 459–480. 10.1016/S1474-4422(18)30499-X 30879893 PMC6459001

[brb33225-bib-0020] Haycock, P. C. , Burgess, S. , Wade, K. H. , Bowden, J. , Relton, C. , & Davey Smith, G. (2016). Best (but oft‐forgotten) practices: The design, analysis, and interpretation of Mendelian randomization studies. American Journal of Clinical Nutrition, 103, 965–978. 10.3945/ajcn.115.118216 26961927 PMC4807699

[brb33225-bib-0021] Hayes, M. T. (2019). Parkinson's disease and Parkinsonism. American Journal of Medicine, 132, 802–807. 10.1016/j.amjmed.2019.03.001 30890425

[brb33225-bib-0022] Hedya, S. A. , Safar, M. M. , & Bahgat, A. K. (2018). Cilostazol Mediated Nurr1 and autophagy enhancement: Neuroprotective activity in rat rotenone PD model. Molecular Neurobiology, 55, 7579–7587. 10.1007/s12035-018-0923-1 29429051

[brb33225-bib-0023] Hollerhage, M. , Moebius, C. , Melms, J. , Chiu, W. H. , Goebel, J. N. , Chakroun, T. , Koeglsperger, T. , Oertel, W. H. , Rosler, T. W. , Bickle, M. , & Höglinger, G. U. (2017). Protective efficacy of phosphodiesterase‐1 inhibition against alpha‐synuclein toxicity revealed by compound screening in LUHMES cells. Scientific Reports, 7, 11469. 10.1038/s41598-017-11664-5 28904388 PMC5597612

[brb33225-bib-0024] Hou, Y. , Dan, X. , Babbar, M. , Wei, Y. , Hasselbalch, S. G. , Croteau, D. L. , & Bohr, V. A. (2019). Ageing as a risk factor for neurodegenerative disease. Nature reviews Neurology, 15, 565–581. 10.1038/s41582-019-0244-7 31501588

[brb33225-bib-0025] Hybiak, J. , Broniarek, I. , Kiryczynski, G. , Los, L. D. , Rosik, J. , Machaj, F. , Slawinski, H. , Jankowska, K. , & Urasinska, E. (2020). Aspirin and its pleiotropic application. European Journal of Pharmacology, 866, 172762. 10.1016/j.ejphar.2019.172762 31669590

[brb33225-bib-0026] Jankovic, J. , & Tan, E. K. (2020). Parkinson's disease: Etiopathogenesis and treatment. Journal of Neurology, Neurosurgery, and Psychiatry, 91, 795–808. 10.1136/jnnp-2019-322338 32576618

[brb33225-bib-0027] Johnson, S. L. , Iannucci, J. , Seeram, N. P. , & Grammas, P. (2020). Inhibiting thrombin improves motor function and decreases oxidative stress in the LRRK2 transgenic *Drosophila melanogaster* model of Parkinson's disease. Biochemical and Biophysical Research Communications, 527, 532–538. 10.1016/j.bbrc.2020.04.068 32423817

[brb33225-bib-0028] Kandil, E. A. , Sayed, R. H. , Ahmed, L. A. , Abd El Fattah, M. A. , & El‐Sayeh, B. M. (2018). Modulatory role of Nurr1 activation and thrombin inhibition in the neuroprotective effects of dabigatran etexilate in rotenone‐induced Parkinson's disease in rats. Molecular Neurobiology, 55, 4078–4089. 10.1007/s12035-017-0636-x 28585189

[brb33225-bib-0029] Krenzlin, H. , Lorenz, V. , Danckwardt, S. , Kempski, O. , & Alessandri, B. (2016). The importance of thrombin in cerebral injury and disease. International Journal of Molecular Sciences, 17(1), 84. 10.3390/ijms17010084 26761005 PMC4730327

[brb33225-bib-0030] Kurilshikov, A. , Medina‐Gomez, C. , Bacigalupe, R. , Radjabzadeh, D. , Wang, J. , Demirkan, A. , Le Roy, C. I. , Raygoza Garay, J. A. , Finnicum, C. T. , Liu, X. , Zhernakova, D. V. , Bonder, M. J. , Hansen, T. H. , Frost, F. , Rühlemann, M. C. , Turpin, W. , Moon, J. Y. , Kim, H. N. , Lüll, K. , … Zhernakova, A. (2021). Large‐scale association analyses identify host factors influencing human gut microbiome composition. Nature Genetics, 53, 156–165. 10.1038/s41588-020-00763-1 33462485 PMC8515199

[brb33225-bib-0031] Majithia, A. , & Bhatt, D. L. (2019). Novel antiplatelet therapies for atherothrombotic diseases. Arteriosclerosis, Thrombosis, and Vascular Biology, 39, 546–557. 10.1161/ATVBAHA.118.310955 30760019 PMC6445601

[brb33225-bib-0032] Page, M. J. , & Pretorius, E. (2022). Platelet behavior contributes to neuropathologies: A focus on Alzheimer's and Parkinson's disease. Seminars in Thrombosis and Hemostasis, 48, 382–404. 10.1055/s-0041-1733960 34624913

[brb33225-bib-0033] Pajares, M. , A, I. R. , Manda, G. , Bosca, L. , & Cuadrado, A. (2020). Inflammation in Parkinson's disease: Mechanisms and therapeutic implications. Cells, 9, 10.3390/cells9071687 PMC7408280 32674367

[brb33225-bib-0034] Paseban, M. , Mohebbati, R. , Niazmand, S. , Sathyapalan, T. , & Sahebkar, A. (2019). Comparison of the neuroprotective effects of aspirin, atorvastatin, captopril and metformin in diabetes mellitus. Biomolecules, 9, 118. 10.3390/biom9040118) 30934759 PMC6523359

[brb33225-bib-0035] Poewe, W. , Seppi, K. , Tanner, C. M. , Halliday, G. M. , Brundin, P. , Volkmann, J. , Schrag, A. E. , & Lang, A. E. (2017). Parkinson disease. Nature Reviews Disease Primers, 3, 17013. 10.1038/nrdp.2017.13 28332488

[brb33225-bib-0036] Rangasamy, S. B. , Dasarathi, S. , Pahan, P. , Jana, M. , & Pahan, K. (2019). Low‐dose aspirin upregulates tyrosine hydroxylase and increases dopamine production in dopaminergic neurons: Implications for Parkinson's disease. Journal of Neuroimmune Pharmacology, 14, 173–187. 10.1007/s11481-018-9808-3 30187283 PMC6401361

[brb33225-bib-0037] San Luciano, M. , Tanner, C. M. , Meng, C. , Marras, C. , Goldman, S. M. , Lang, A. E. , Tolosa, E. , Schule, B. , Langston, J. W. , Brice, A. , Corvol, J. C. , Goldwurm, S. , Klein, C. , Brockman, S. , Berg, D. , Brockmann, K. , Ferreira, J. J. , Tazir, M. , Mellick, G. D. , … Michael, J. , Fox Foundation LRRK2 Cohort Consortium . (2020). Nonsteroidal anti‐inflammatory use and LRRK2 Parkinson's disease penetrance. Movement Disorders, 35, 1755–1764. 10.1002/mds.28189 32662532 PMC7572560

[brb33225-bib-0038] Skrivankova, V. W. , Richmond, R. C. , Woolf, B. A. R. , Yarmolinsky, J. , Davies, N. M. , Swanson, S. A. , VanderWeele, T. J. , Higgins, J. P. T. , Timpson, N. J. , Dimou, N. , Langenberg, C. , Golub, R. M. , Loder, E. W. , Gallo, V. , Tybjaerg‐Hansen, A. , Davey Smith, G. , Egger, M. , & Richards, J. B. (2021). Strengthening the reporting of observational studies in epidemiology using Mendelian Randomization: The STROBE‐MR statement. JAMA, 326, 1614–1621. 10.1001/jama.2021.18236 34698778

[brb33225-bib-0039] Smith, G. D. , & Ebrahim, S. (2004). Mendelian randomization: Prospects, potentials, and limitations. International Journal of Epidemiology, 33, 30–42. 10.1093/ije/dyh132 15075143

[brb33225-bib-0040] Starhof, C. , Hejl, A. M. , Korbo, L. , Winge, K. , & Friis, S. (2020). Risk of multiple system atrophy and the use of anti‐inflammatory drugs: A Danish Register‐based case‐control study. Neuroepidemiology, 54, 58–63. 10.1159/000503003 31661696

[brb33225-bib-0041] Subramaniam, S. R. , & Chesselet, M. F. (2013). Mitochondrial dysfunction and oxidative stress in Parkinson's disease. Progress in Neurobiology, 106–107, 17–32. 10.1016/j.pneurobio.2013.04.004 PMC3742021 23643800

[brb33225-bib-0042] Sun, Y. , Li, Y. , & Zhang, J. (2022). The causal relationship between psoriasis, psoriatic arthritis, and inflammatory bowel diseases. Scientific Reports, 12, 20526. 10.1038/s41598-022-24872-5 36443384 PMC9705442

[brb33225-bib-0043] Tansey, M. G. , Wallings, R. L. , Houser, M. C. , Herrick, M. K. , Keating, C. E. , & Joers, V. (2022). Inflammation and immune dysfunction in Parkinson disease. Nature Reviews Immunology, 22, 657–673. 10.1038/s41577-022-00684-6 PMC8895080 35246670

[brb33225-bib-0044] Tao, Y. , Sun, Y. , Lv, S. , Xia, W. , Zhao, K. , Xu, Q. , Zhao, Q. , He, L. , Le, W. , Wang, Y. , Liu, C. , & Li, D. (2022). Heparin induces alpha‐synuclein to form new fibril polymorphs with attenuated neuropathology. Nature Communications, 13, 4226. 10.1038/s41467-022-31790-7 PMC9307803 35869048

[brb33225-bib-0045] Thrash‐Williams, B. , Karuppagounder, S. S. , Bhattacharya, D. , Ahuja, M. , Suppiramaniam, V. , & Dhanasekaran, M. (2016). Methamphetamine‐induced dopaminergic toxicity prevented owing to the neuroprotective effects of salicylic acid. Life Sciences, 154, 24–29. 10.1016/j.lfs.2016.02.072 26926078

[brb33225-bib-0046] Trist, B. G. , Hare, D. J. , & Double, K. L. (2019). Oxidative stress in the aging substantia nigra and the etiology of Parkinson's disease. Aging Cell, 18, e13031. 10.1111/acel.13031 31432604 PMC6826160

[brb33225-bib-0047] Verbanck, M. , Chen, C. Y. , Neale, B. , & Do, R. (2018). Detection of widespread horizontal pleiotropy in causal relationships inferred from Mendelian randomization between complex traits and diseases. Nature Genetics, 50, 693–698. 10.1038/s41588-018-0099-7 29686387 PMC6083837

[brb33225-bib-0048] Vijiaratnam, N. , Simuni, T. , Bandmann, O. , Morris, H. R. , & Foltynie, T. (2021). Progress towards therapies for disease modification in Parkinson's disease. Lancet Neurology, 20, 559–572. 10.1016/S1474-4422(21)00061-2 34146514

[brb33225-bib-0049] Wu, Y. , Byrne, E. M. , Zheng, Z. , Kemper, K. E. , Yengo, L. , Mallett, A. J. , Yang, J. , Visscher, P. M. , & Wray, N. R. (2019). Genome‐wide association study of medication‐use and associated disease in the UK Biobank. Nature Communications, 10(1), 1891. 10.1038/s41467-019-09572-5 PMC6478889 31015401

