# Supplementary material for: Association between 23 drugs and Parkinson's disease: A two‐sample Mendelian randomization study
Source: Brain Behav. 2023 Aug 31;13(11):e3225. doi: 10.1002/brb3.3225 (PMC10636399; doi:10.1002/brb3.3225)
Supplement: Supplementary file 2 — Supporting Information [file BRB3-13-e3225-s001.docx]

**STROBE-MR checklist of recommended items to address in reports of Mendelian randomization studies**^1^ ^2^

| **Item No.** | **Section** | **Checklist item** | **Page No.** | **Relevant text from manuscript** |
| --- | --- | --- | --- | --- |
| 1 | **TITLE and ABSTRACT** | Indicate Mendelian randomization (MR) as the study’s design in the title and/or the abstract if that is a main purpose of the study | 1,2 | Title：  Association between 23 drugs and Parkinson's disease: A Two-Sample Mendelian Randomization Study  Abstract：  Background: Parkinson's disease (PD) is a common degenerative nervous system disease. At present, there are certain limitations in various treatment options aimed at preventing or delaying the progression of Parkinson's disease (PD). Therefore, the exploration of new drugs for PD is beneficial. This study analyzed the relationship between 23 drugs and PD. These drugs have already been approved for the treatment of different diseases, such as Salicylic acid and derivatives (collectively called Salicylates, e.g. aspirin, used for fever and pain relief), Antithrombotic agents (e.g. warfarin, aspirin, used for preventing thrombotic events), and Vasodilators used in cardiac disease (e.g. nitroglycerin, used to alleviate angina). Mendelian randomization (MR) analysis can be used to explore the association between drugs and diseases. In this study, MR analysis was adopted to investigate the causal relationship between 23 drugs and PD.  Methods: The GWAS data for the 23 drugs were obtained from the UK Biobank (UKB) project, while the GWAS data for PD were sourced from FinnGen. Single-Nucleotide Polymorphisms (SNPs) were selected as Instrumental Variables (IVs) for the analysis. We first performed a series of quality control steps (including MR-PRESSO) to select the appropriate SNPs. Two-sample MR analysis was performed using five different methods, including Inverse Variance Weighting (IVW) with random-effects model, Weighted median, MR-Egger, Simple model, and Weighted model. At the same time, sensitivity analysis was carried out using the MR-Egger and Cochran's Q test to ensure the authenticity and reliability of the results.  Results: In MR-PRESSO, Salicylates and Antithrombotic agents showed statistically significant associations with PD, respectively. In the main MR Analysis (IVW), there was a negative causal relationship between Salicylates and PD (OR=0.73, 95% CI=0.54-0.98, P=0.039). Similarly, there was a negative causal relationship between Antithrombotic agents and PD (OR=0.70, 95%CI=0.52-0.96, P=0.027). No statistically significant association was found between the remaining 21 drugs and PD.  Conclusion: This MR study demonstrated that Salicylates and Antithrombotic agents can reduce the risk of PD, thus providing a novel avenue for future drug exploration in PD. |
|  | **INTRODUCTION** |  |  |  |
| 2 | **Background** | Explain the scientific background and rationale for the reported study. What is the exposure? Is a potential causal relationship between exposure and outcome plausible? Justify why MR is a helpful method to address the study question | 2 | Current treatments have limitations in preventing or delaying PD progression. Hence, exploring therapeutic drugs for PD through new methods may be beneficial. Mendelian randomization (MR) analysis, which employs genetic variation as instrumental variables (IVs), is a method for inferring causal relationships between exposure and outcome. MR analysis can overcome the effects of confounding factors, such as behavioral and environmental factors. Moreover, it can provide reliable evidence for causal relationships between risk factors and diseases, while guiding the direction of clinical trials and drug development.  Mendelian randomization (MR) analysis, which employs genetic variation as instrumental variables (IVs), is a method for inferring causal relationships between exposure and outcome. MR analysis can overcome the effects of confounding factors, such as behavioral and environmental factors. Moreover, it can provide reliable evidence for causal relationships between risk factors and diseases, while guiding the direction of clinical trials and drug development. |
| 3 | **Objectives** | State specific objectives clearly, including pre-specified causal hypotheses (if any). State that MR is a method that, under specific assumptions, intends to estimate causal effects | 2,3 | This study employs Two-sample MR to assess causal relationships between multiple drugs and PD, utilizing the latest drug genome-wide association study (GWAS) data that covers a wide range of populations, and evaluating the reliability of MR results. |
|  | **METHODS** |  |  |  |
| 4 | **Study design and data sources** | Present key elements of the study design early in the article. Consider including a table listing sources of data for all phases of the study. For each data source contributing to the analysis, describe the following: | 3 | This is an MR study investigating the causal relationship between 23 drugs and Parkinson's disease (PD). The MR study relies on the strict adherence to three assumptions: (1) the relevance assumption, where IVs should be strongly associated with the exposure; (2) the independence assumption, where the effect of IVs on the outcome can only be mediated through the exposure; (3) the exclusion restriction assumption, where IVs should not have a direct association with the outcome. Moreover, we have followed the recommendations of Strengthening the Reporting of Observational Studies in Epidemiology Using Mendelian Randomization (STROBE-MR) to ensure the replicability of our study.  The R package TwoSampleMR was utilized for conducting the MR analysis, while the R package MR-PRESSO was employed for performing MR-PRESSO. All the aforementioned analyses were carried out on R software version 4.2.1. |
|  | a) | Setting: Describe the study design and the underlying population, if possible. Describe the setting, locations, and relevant dates, including periods of recruitment, exposure, follow-up, and data collection, when available. | 3 | Our study sample was obtained from the UK Biobank (UKB) project (<https://www.ukbiobank.ac.uk/>), in which 502,616 participants (about 54% females) had medical records at their first UKB assessment visit.  For the outcome dataset, GWAS data for PD were obtained from FinnGen (<https://Finngen.gitbook.io/documentation/>) and included 3,767 cases and 338,732 controls.  More summary statistics about the exposure and outcome are presented in the Supplementary file S1-S2. |
|  | b) | Participants: Give the eligibility criteria, and the sources and methods of selection of participants. Report the sample size, and whether any power or sample size calculations were carried out prior to the main analysis | 3 | Our study sample was obtained from the UK Biobank (UKB) project (<https://www.ukbiobank.ac.uk/>), in which 502,616 participants (about 54% females) had medical records at their first UKB assessment visit.  For the outcome dataset, GWAS data for PD were obtained from FinnGen (<https://Finngen.gitbook.io/documentation/>) and included 3,767 cases and 338,732 controls.  More summary statistics about the exposure and outcome are presented in the Supplementary file S1-S2. |
|  | c) | Describe measurement, quality control and selection of genetic variants | 4 | We selected SNPs with a genome-wide association (p< 5E-08), with independent inheritance (r^2^< 0.01), and without linkage disequilibrium (LD) in summary statistics. We also calculated the F statistic for each exposure to avoid bias due to weak genetic instruments. IVs with an F statistic of less than 10 were excluded and were often labeled as “weak instruments”  Since subsequent analysis required at least 4 SNPs as instrumental variables, we relaxed the p-value threshold to P<5E-07 for screening drug GWAS with insufficient instrumental variables after normalization. If that's not enough, the threshold was further relaxed to P<5E-06. Moreover, the outliers of missing data were excluded. |
|  | d) | For each exposure, outcome, and other relevant variables, describe methods of assessment and diagnostic criteria for diseases | / | / |
|  | e) | Provide details of ethics committee approval and participant informed consent, if relevant | / | / |
| 5 | **Assumptions** | Explicitly state the three core IV assumptions for the main analysis (relevance, independence and exclusion restriction) as well assumptions for any additional or sensitivity analysis | 3,4 | The MR study relies on the strict adherence to three assumptions: (1) the relevance assumption, where IVs should be strongly associated with the exposure; (2) the independence assumption, where the effect of IVs on the outcome can only be mediated through the exposure; (3) the exclusion restriction assumption, where IVs should not have a direct association with the outcome.  We conducted the MR-Egger regression to evaluate the possibility of horizontal pleiotropy. The intercept term of the MR-Egger regression shows the mean pleiotropic effect IV. To assess the heterogeneity of the effects we used Cochran's Q test with IVW and MR-Egger, and P< 0.05 determined by Cochran's Q test was considered heterogeneous. The Leave-one-out sensitivity analysis was performed to test the robustness of the association results by removing studies individually. |
| 6 | **Statistical methods: main analysis** | Describe statistical methods and statistics used |  |  |
|  | a) | Describe how quantitative variables were handled in the analyses (i.e., scale, units, model) | / | / |
|  | b) | Describe how genetic variants were handled in the analyses and, if applicable, how their weights were selected | 3,4 | We selected SNPs with a genome-wide association (p< 5E-08), with independent inheritance (r2< 0.01), and without linkage disequilibrium (LD) in summary statistics. We also calculated the F statistic for each exposure to avoid bias due to weak genetic instruments. IVs with an F statistic of less than 10 were excluded and were often labeled as “weak instruments”  Since subsequent analysis required at least 4 SNPs as instrumental variables, we relaxed the p-value threshold to P<5E-07 for screening drug GWAS with insufficient instrumental variables after normalization. If that's not enough, the threshold was further relaxed to P<5E-06. Moreover, the outliers of missing data were excluded. |
|  | c) | Describe the MR estimator (e.g. two-stage least squares, Wald ratio) and related statistics. Detail the included covariates and, in case of two-sample MR, whether the same covariate set was used for adjustment in the two samples | 4 | After identifying the genetic instruments for each exposure, genetic variants associated with drugs were selected as genetic instruments for each exposure. We first applied MR-PRESSO to detect and correct for any outliers reflecting likely pleiotropic biases for all reported results. Two-sample MR analysis was performed using five different methods, including Inverse Variance Weighted (IVW), Weighted median, MR-Egger, Simple mode, and Weighted mode. Each method makes different assumptions on the validity of IVs, but the IVW method is generally considered the most reliable. IVW was used as our principal model, which accounts for heterogeneity in the variant-specific causal estimates. The other methods were used as complementary or to observe if their results were consistent with the direction of IVW. Finally, a forest plot was used for visualization. |
|  | d) | Explain how missing data were addressed | 4 | Moreover, the outliers of missing data were excluded. |
|  | e) | If applicable, indicate how multiple testing was addressed | / | / |
| 7 | **Assessment of assumptions** | Describe any methods or prior knowledge used to assess the assumptions or justify their validity | 3,4 | To satisfy the three strict assumptions mentioned earlier, we performed a series of quality control steps to select suitable SNPs. We selected SNPs with a genome-wide association (p< 5E-08), with independent inheritance (r2< 0.01), and without linkage disequilibrium (LD) in summary statistics. We also calculated the F statistic for each exposure to avoid bias due to weak genetic instruments. IVs with an F statistic of less than 10 were excluded and were often labeled as “weak instruments”  We conducted the MR-Egger regression to evaluate the possibility of horizontal pleiotropy. The intercept term of the MR-Egger regression shows the mean pleiotropic effect IV. To assess the heterogeneity of the effects we used Cochran's Q test with IVW and MR-Egger, and P< 0.05 determined by Cochran's Q test was considered heterogeneous. The Leave-one-out sensitivity analysis was performed to test the robustness of the association results by removing studies individually. |
| 8 | **Sensitivity analyses and additional analyses** | Describe any sensitivity analyses or additional analyses performed (e.g. comparison of effect estimates from different approaches, independent replication, bias analytic techniques, validation of instruments, simulations) | 4 | In this study, sensitivity analyses were performed using different methods. We conducted the MR-Egger regression to evaluate the possibility of horizontal pleiotropy. The intercept term of the MR-Egger regression shows the mean pleiotropic effect IV. To assess the heterogeneity of the effects we used Cochran's Q test with IVW and MR-Egger, and P< 0.05 determined by Cochran's Q test was considered heterogeneous. The Leave-one-out sensitivity analysis was performed to test the robustness of the association results by removing studies individually. |
| 9 | **Software and pre-registration** |  |  |  |
|  | a) | Name statistical software and package(s), including version and settings used | 3 | The R package TwoSampleMR was utilized for conducting the MR analysis, while the R package MR-PRESSO was employed for performing MR-PRESSO. All the aforementioned analyses were carried out on R software version 4.2.1. |
|  | b) | State whether the study protocol and details were pre-registered (as well as when and where) | / | / |
|  | **RESULTS** |  |  |  |
| 10 | **Descriptive data** |  |  |  |
|  | a) | Report the numbers of individuals at each stage of included studies and reasons for exclusion. Consider use of a flow diagram | 3 | Our study sample was obtained from the UK Biobank (UKB) project (<https://www.ukbiobank.ac.uk/>), in which 502,616 participants (about 54% females) had medical records at their first UKB assessment visit.  For the outcome dataset, GWAS data for PD were obtained from FinnGen (<https://Finngen.gitbook.io/documentation/>) and included 3,767 cases and 338,732 controls.  More summary statistics about the exposure and outcome are presented in the Supplementary file S1-S2. |
|  | b) | Report summary statistics for phenotypic exposure(s), outcome(s), and other relevant variables (e.g. means, SDs, proportions) | 3 | Our study sample was obtained from the UK Biobank (UKB) project (<https://www.ukbiobank.ac.uk/>), in which 502,616 participants (about 54% females) had medical records at their first UKB assessment visit.  For the outcome dataset, GWAS data for PD were obtained from FinnGen (<https://Finngen.gitbook.io/documentation/>) and included 3,767 cases and 338,732 controls.  More summary statistics about the exposure and outcome are presented in the Supplementary file S1-S2. |
|  | c) | If the data sources include meta-analyses of previous studies, provide the assessments of heterogeneity across these studies | / | / |
|  | d) | For two-sample MR:  i.  Provide justification of the similarity of the genetic variant-exposure associations between the exposure and outcome samples  ii.  Provide information on the number of individuals who overlap between the exposure and outcome studies | 3 | In this study, the sample for the GWAS of 23 drugs primarily comes from the UK Biobank (UKB), while the sample for the GWAS of Parkinson's disease (PD) comes from FinnGen. It implied that the GWAS data for exposure and outcomes were derived from two largely independent samples, making sample overlap and its impact on the study results potentially negligible.  In addition, both samples consist of individuals of European ancestry, so it can be considered that there was the similarity of the genetic variant-exposure associations between the exposure and outcome samples. |
| 11 | **Main results** |  |  |  |
|  | a) | Report the associations between genetic variant and exposure, and between genetic variant and outcome, preferably on an interpretable scale | / | / |
|  | b) | Report MR estimates of the relationship between exposure and outcome, and the measures of uncertainty from the MR analysis, on an interpretable scale, such as odds ratio or relative risk per SD difference | 5 | After identifying and removing abnormal SNPs, the results of MR-PRESSO described a statistically significant causal relationship between Salicylates (P=0.017), Antithrombotic agents (P=0.022), and PD (Supplementary file S3). In MR analysis, IVW showed that both two factors were negatively associated with the incidence of PD. As shown in Figure 1 and the Supplementary file S3, Salicylates reduced the risk of PD (OR=0.73, 95% CI=0.54-0.98, P=0.039), and the directions of β values of the other four methods are consistent with IVW. In addition, MR analysis also demonstrated the therapeutic effect of Antithrombotic agents on PD (OR=0.70, 95%CI=0.52-0.96, P=0.027), and the direction of β value of the five MR methods was consistent, which confirmed the robustness and reliability of the results. However, no causal relationship between other drugs and PD was found. Details are listed in the Supplementary file S3. |
|  | c) | If relevant, consider translating estimates of relative risk into absolute risk for a meaningful time period | / | / |
|  | d) | Consider plots to visualize results (e.g. forest plot, scatterplot of associations between genetic variants and outcome versus between genetic variants and exposure) | 5 | Figure 1 and 4 |
| 12 | **Assessment of assumptions** |  |  |  |
|  | a) | Report the assessment of the validity of the assumptions | 5 | All included SNPs adhere to the criteria of independent inheritance (r2 < 0.01), no linkage disequilibrium (LD), and F>10 ("weak instruments" were excluded). For the inclusion of other exposed SNPs, see the Supplementary file S3 for details. |
|  | b) | Report any additional statistics (e.g., assessments of heterogeneity across genetic variants, such as *I^2^*, Q statistic or E-value) | 5 | We performed MR-Egger intercept tests to evaluate the possibility of horizontal pleiotropy, and no significant horizontal pleiotropy was found in the association of Salicylates with PD (intercept=0.014, P=0.707). Similarly, MR-Egger showed that there was no horizontal pleiotropy between Antithrombotic agents and PD (P = 0.499), which further proved the reliability of our causal inference results. Bias from horizontal pleiotropies could be largely ruled out by using Leave-one-out analysis, which shows that our MR results are stable and not driven by any single SNP (Figure 2). In Cochran's Q test for heterogeneity, both IVW (Q=3.787, P=0.876) and MR-Egger (Q=3.633, P= 0.821) showed there was no heterogeneity among SNPs of Salicylates. In addition, Cochran's Q test between Antithrombotic agents and PD also shows no heterogeneity (P>0.05), indicating that the IVW results of the multiplicative random effects method should be the first choice. Funnel plot and scatter plot were used for visualization (Figures 3 and 4).  Finally, the summary results of MR estimation and sensitivity analysis of all drugs and PD are given in the Supplementary file S3. Overall, the significance of the causal relationships between Salicylates and PD, as well as Antithrombotic agents and PD, were repeated by MR-PRESSO and IVW and passed the pleiotropic test. It was found in our MR study that Salicylates and Antithrombotic agents can reduce the risk of PD. |
| 13 | **Sensitivity analyses and additional analyses** |  |  |  |
|  | a) | Report any sensitivity analyses to assess the robustness of the main results to violations of the assumptions | 5 | We performed MR-Egger intercept tests to evaluate the possibility of horizontal pleiotropy, and no significant horizontal pleiotropy was found in the association of Salicylates with PD (intercept=0.014, P=0.707). Similarly, MR-Egger showed that there was no horizontal pleiotropy between Antithrombotic agents and PD (P = 0.499), which further proved the reliability of our causal inference results. Bias from horizontal pleiotropies could be largely ruled out by using Leave-one-out analysis, which shows that our MR results are stable and not driven by any single SNP (Figure 2). In Cochran's Q test for heterogeneity, both IVW (Q=3.787, P=0.876) and MR-Egger (Q=3.633, P= 0.821) showed there was no heterogeneity among SNPs of Salicylates. In addition, Cochran's Q test between Antithrombotic agents and PD also shows no heterogeneity (P>0.05), indicating that the IVW results of the multiplicative random effects method should be the first choice. Funnel plot and scatter plot were used for visualization (Figures 3 and 4).  Finally, the summary results of MR estimation and sensitivity analysis of all drugs and PD are given in the Supplementary file S3. Overall, the significance of the causal relationships between Salicylates and PD, as well as Antithrombotic agents and PD, were repeated by MR-PRESSO and IVW and passed the pleiotropic test. It was found in our MR study that Salicylates and Antithrombotic agents can reduce the risk of PD. |
|  | b) | Report results from other sensitivity analyses or additional analyses | / | / |
|  | c) | Report any assessment of direction of causal relationship (e.g., bidirectional MR) | 5 | After identifying and removing abnormal SNPs, the results of MR-PRESSO described a statistically significant causal relationship between Salicylates (P=0.017), Antithrombotic agents (P=0.022), and PD (Supplementary file S3). In MR analysis, IVW showed that both two factors were negatively associated with the incidence of PD. As shown in Figure 1 and the Supplementary file S3, Salicylates reduced the risk of PD (OR=0.73, 95% CI=0.54-0.98, P=0.039), and the directions of β values of the other four methods are consistent with IVW. In addition, MR analysis also demonstrated the therapeutic effect of Antithrombotic agents on PD (OR=0.70, 95%CI=0.52-0.96, P=0.027), and the direction of β value of the five MR methods was consistent, which confirmed the robustness and reliability of the results. However, no causal relationship between other drugs and PD was found. Details are listed in the Supplementary file S3. |
|  | d) | When relevant, report and compare with estimates from non-MR analyses | / | / |
|  | e) | Consider additional plots to visualize results (e.g., leave-one-out analyses) | 5 | Figure 2 and 3 |
|  | **DISCUSSION** |  |  |  |
| 14 | **Key results** | Summarize key results with reference to study objectives | 5,6 | Based on 23 exposure GWAS datasets and 1 outcome GWAS dataset, this MR analysis examined the relationship between 23 drugs and PD and found a statistically negative correlation between Salicylates and PD, as well as between Antithrombotic agents and PD, indicating that both drugs can reduce the risk of PD. No significant association was found between the other 21 drugs and PD. The results of this analysis provide support for the causal relationship between Salicylates and PD, and Antithrombotic agents and PD, and offer new insights for the development of PD drug treatments. |
| 15 | **Limitations** | Discuss limitations of the study, taking into account the validity of the IV assumptions, other sources of potential bias, and imprecision. Discuss both direction and magnitude of any potential bias and any efforts to address them | 7 | However, our study also has some limitations. The GWAS data for PD and the 23 drugs are predominantly derived from individuals of European ancestry, with no representation of other ethnic populations. Hence, caution must be exercised when extrapolating our MR analysis outcomes to other groups as it may only be generalizable to European ancestry populations. Furthermore, while Two-sample MR analysis serves as a valuable tool for assessing the causal relationship between drugs and PD, it has certain shortcomings, as it only furnishes estimates of hypothetical causal associations. Consequently, further investigations are imperative to authenticate the direct causal impact of these 23 drugs on PD. |
| 16 | **Interpretation** |  |  |  |
|  | a) | Meaning: Give a cautious overall interpretation of results in the context of their limitations and in comparison with other studies | 6,7 | Oxidative stress and inflammation are key factors in the progression of PD. When there is oxidative stress within cells, the accumulation of Reactive Oxygen Species (ROS) can lead to oxidative damage, thus damaging neurons. Inflammation is also commonly present in the progression of PD, and various pathological and physiological processes such as activation of microglial cells and regulation of the immune system can cause inflammation, thereby affecting the nervous syste. Oxidative stress can cause oxidative damage, stimulate the production of inflammation, and the presence of inflammatory factors can exacerbate oxidative damage, leading to a vicious cycle.  Aspirin inhibits the enzyme activity of cyclooxygenase (COX), which affects the generation of prostaglandins (PGs), thereby inhibiting inflammation. At the same time, aspirin also has an antioxidative stress effect, which can prevent oxidative damage. Therefore, aspirin may affect PD by influencing these two factors. However, it should be noted that the conclusions of current studies are not consistent. According to a retrospective investigation, the utilization of aspirin has been purportedly linked with a considerably heightened vulnerability to PD. Also, there was no statistically significant correlation between low-dose aspirin and the risk of multiple system atrophy (MSA, a rare atypical form of PD). A case-control study also found no statistically significant association between the long-term use of aspirin and the incidence of PD. Therefore, more research is needed to determine the relationship between Salicylates and PD.  Inhibition of platelet aggregation and thrombin is an important means of anti-thrombosis. Inflammation promotes platelet aggregation in cerebral microvasculature, while soluble factors released by activated platelets in turn promote the development of inflammation. Some antiplatelet drugs show promising results in the treatment of PD. Aspirin irreversibly inhibits platelet COX enzyme, thereby inhibiting platelet aggregation mediated by TXA2. Aspirin also exhibits anti-inflammatory and antioxidant effects, and has been suggested to be beneficial for PD in some studies. Cilostazol can increase the expression of Nurr1 in mice, protecting the integrity of dopaminergic neurons and reducing inflammation by suppressing NF-κB and its downstream effectors TNF-α and IL-1β. In a 2017 study, Dipyridamole was shown to have potential value in protecting neurons in PD patients by reducing levels of lactate dehydrogenase (LDH) in LUHMES cells with overexpression of α-synuclein. On the other hand, antiplatelet drugs can also be used to control cardiovascular diseases (common complications of PD) and inhibit thrombus formation, thereby improving patient prognosis.  Thrombin induces the expression of pro-inflammatory cytokines such as NO, IL-1β, IL-6, and TNF-α by activating PAR, promoting inflammation, and driving the progression of neurodegenerative diseases. In PD rats induced by rotenone, dabigatran etexilate inhibited thrombin levels and reduced the inflammation level in the substantia nigra, thus exerting a neuroprotective effect. In another in vitro experiment, dabigatran inhibited thrombin and reduced the expression of NOX4, iNOS, and SOD in fruit flies, suggesting that thrombin inhibition can reduce oxidative stress, which may be beneficial for PD patients. In vitro, Heparin induced the formation of new α-Synuclein complexes, thereby reducing the pathogenicity of amyloid fibrils on neurons. Direct oral anticoagulants (DOACs) can interrupt thrombin-induced neurotoxicity and neuroinflammation, thus protecting PD patients. These findings suggest that Antithrombotic agents have great potential in the treatment of PD. |
|  | b) | Mechanism: Discuss underlying biological mechanisms that could drive a potential causal relationship between the investigated exposure and the outcome, and whether the gene-environment equivalence assumption is reasonable. Use causal language carefully, clarifying that IV estimates may provide causal effects only under certain assumptions | 6,7 | Oxidative stress and inflammation are key factors in the progression of PD. When there is oxidative stress within cells, the accumulation of Reactive Oxygen Species (ROS) can lead to oxidative damage, thus damaging neurons. Inflammation is also commonly present in the progression of PD, and various pathological and physiological processes such as activation of microglial cells and regulation of the immune system can cause inflammation, thereby affecting the nervous syste. Oxidative stress can cause oxidative damage, stimulate the production of inflammation, and the presence of inflammatory factors can exacerbate oxidative damage, leading to a vicious cycle.  Aspirin inhibits the enzyme activity of cyclooxygenase (COX), which affects the generation of prostaglandins (PGs), thereby inhibiting inflammation. At the same time, aspirin also has an antioxidative stress effect, which can prevent oxidative damage. Therefore, aspirin may affect PD by influencing these two factors. However, it should be noted that the conclusions of current studies are not consistent. According to a retrospective investigation, the utilization of aspirin has been purportedly linked with a considerably heightened vulnerability to PD. Also, there was no statistically significant correlation between low-dose aspirin and the risk of multiple system atrophy (MSA, a rare atypical form of PD). A case-control study also found no statistically significant association between the long-term use of aspirin and the incidence of PD. Therefore, more research is needed to determine the relationship between Salicylates and PD.  Inhibition of platelet aggregation and thrombin is an important means of anti-thrombosis. Inflammation promotes platelet aggregation in cerebral microvasculature, while soluble factors released by activated platelets in turn promote the development of inflammation. Some antiplatelet drugs show promising results in the treatment of PD. Aspirin irreversibly inhibits platelet COX enzyme, thereby inhibiting platelet aggregation mediated by TXA2. Aspirin also exhibits anti-inflammatory and antioxidant effects, and has been suggested to be beneficial for PD in some studies. Cilostazol can increase the expression of Nurr1 in mice, protecting the integrity of dopaminergic neurons and reducing inflammation by suppressing NF-κB and its downstream effectors TNF-α and IL-1β. In a 2017 study, Dipyridamole was shown to have potential value in protecting neurons in PD patients by reducing levels of lactate dehydrogenase (LDH) in LUHMES cells with overexpression of α-synuclein. On the other hand, antiplatelet drugs can also be used to control cardiovascular diseases (common complications of PD) and inhibit thrombus formation, thereby improving patient prognosis.  Thrombin induces the expression of pro-inflammatory cytokines such as NO, IL-1β, IL-6, and TNF-α by activating PAR, promoting inflammation, and driving the progression of neurodegenerative diseases. In PD rats induced by rotenone, dabigatran etexilate inhibited thrombin levels and reduced the inflammation level in the substantia nigra, thus exerting a neuroprotective effect. In another in vitro experiment, dabigatran inhibited thrombin and reduced the expression of NOX4, iNOS, and SOD in fruit flies, suggesting that thrombin inhibition can reduce oxidative stress, which may be beneficial for PD patients. In vitro, Heparin induced the formation of new α-Synuclein complexes, thereby reducing the pathogenicity of amyloid fibrils on neurons. Direct oral anticoagulants (DOACs) can interrupt thrombin-induced neurotoxicity and neuroinflammation, thus protecting PD patients. These findings suggest that Antithrombotic agents have great potential in the treatment of PD. |
|  | c) | Clinical relevance: Discuss whether the results have clinical or public policy relevance, and to what extent they inform effect sizes of possible interventions | 5,6 | The results of this analysis provide support for the causal relationship between Salicylates and PD, and Antithrombotic agents and PD, and offer new insights for the development of PD drug treatments. |
| 17 | **Generalizability** | Discuss the generalizability of the study results (a) to other populations, (b) across other exposure periods/timings, and (c) across other levels of exposure | 7 | However, our study also has some limitations. The GWAS data for PD and the 23 drugs are predominantly derived from individuals of European ancestry, with no representation of other ethnic populations. Hence, caution must be exercised when extrapolating our MR analysis outcomes to other groups as it may only be generalizable to European ancestry populations. |
|  | **OTHER INFORMATION** |  |  |  |
| 18 | **Funding** | Describe sources of funding and the role of funders in the present study and, if applicable, sources of funding for the databases and original study or studies on which the present study is based | 7 | The present study was supported by the National Natural Science Foundation of China, Youth Science Foundation Project (82101327), Natural Science Foundation of Guangdong Province, General Project (2022A1515012362), Regional Consolidated Fund - Youth Fund Project (2019A1515110150). |
| 19 | **Data and data sharing** | Provide the data used to perform all analyses or report where and how the data can be accessed, and reference these sources in the article. Provide the statistical code needed to reproduce the results in the article, or report whether the code is publicly accessible and if so, where | 7 | The summary GWAS dataset of 23 drugs was obtained from the UK Biobank (UKB) project (<https://pubmed.ncbi.nlm.nih.gov/31015401/>). GWAS dataset for PD was obtained from FinnGen (<https://storage.googleapis.com/finngen-public-data-r8/summary_stats/finngen_R8_G6_PARKINSON.gz>). The results of the analysis can be found in the manuscript and supplementary documents. |
| 20 | **Conflicts of Interest** | All authors should declare all potential conflicts of interest | 7 | We have no competing interests. |

This checklist is copyrighted by the Equator Network under the Creative Commons Attribution 3.0 Unported (CC BY 3.0) license.

1. Skrivankova VW, Richmond RC, Woolf BAR, Yarmolinsky J, Davies NM, Swanson SA, et al. Strengthening the Reporting of Observational Studies in Epidemiology using Mendelian Randomization (STROBE-MR) Statement. JAMA. 2021;under review.

2. Skrivankova VW, Richmond RC, Woolf BAR, Davies NM, Swanson SA, VanderWeele TJ, et al. Strengthening the Reporting of Observational Studies in Epidemiology using Mendelian Randomisation (STROBE-MR): Explanation and Elaboration. BMJ. 2021;375:n2233.
